# Off-Target Effects of Antidepressants on Vascular Function and Structure

**DOI:** 10.3390/biomedicines10010056

**Published:** 2021-12-28

**Authors:** Anna Dimoula, Dimitrios Fotellis, Evmorfia Aivalioti, Dimitrios Delialis, Alexia Polissidis, Raphael Patras, Nikolaos Kokras, Kimon Stamatelopoulos

**Affiliations:** 1 Department of Clinical Therapeutics, Alexandra Hospital, Medical School, National and Kapodistrian University of Athens, 80 Vas. Sofias Str., 11528 Athens, Greece; dimoulanna@gmail.com (A.D.); fotellisdimitris@gmail.com (D.F.); morfoaival5@gmail.com (E.A.); dimitrisdelialis@gmail.com (D.D.); raphaelpatras@gmail.com (R.P.); 2 Department of Pharmacology, Medical School, National and Kapodistrian University of Athens, 11527 Athens, Greece; apolissidis@bioacademy.gr (A.P.); nkokras@med.uoa.gr (N.K.); 3 Center of Clinical, Experimental Surgery and Translational Research, Biomedical Research Foundation of the Academy of Athens (BRFAA), 4 Soranou Efesiou St., 11527 Athens, Greece; 4 First Department of Psychiatry, Eginition Hospital, Medical School, National and Kapodistrian University of Athens, 11527 Athens, Greece; 5 Biosciences Institute, Faculty of Medical Sciences, Newcastle University, Newcastle Upon Tyne NE2 4HH, UK

**Keywords:** antidepressants, cardiovascular, hypertension, atherosclerosis, FMD (flow mediated dilation), PWV (pulse wave velocity)

## Abstract

Depression emerges as a risk factor for cardiovascular disease, and it is thought that successful antidepressant treatment may reduce such a risk. Therefore, antidepressant treatment embodies a potential preventive measure to reduce cardiovascular events in patients with depression. Accumulating evidence indicates that antidepressants have off-target effects on vascular dysfunction and in the early stages of atherosclerosis, which form the basis for cardiovascular disease (CVD) pathogenesis. In this context, we performed a thorough review of the evidence pertaining to the effects of different classes of antidepressant medications on hemodynamic and early atherosclerosis markers. The preclinical and clinical evidence reviewed revealed a preponderance of studies assessing selective serotonin reuptake inhibitors (SSRI), whereas other classes of antidepressants are less well-studied. Sufficient evidence supports a beneficial effect of SSRIs on vascular inflammation, endothelial function, arterial stiffening, and possibly delaying carotid atherosclerosis. In clinical studies, dissecting the hypothesized direct beneficial antidepressant effect of SSRIs on endothelial health from the global improvement upon remission of depression has proven to be difficult. However, preclinical studies armed with appropriate control groups provide evidence of molecular mechanisms linked to endothelial function that are indeed modulated by antidepressants. This suggests at least a partial direct action on vascular integrity. Further research on endothelial markers should focus on the effect of antidepressants on treatment responders versus non-responders in order to better ascertain the possible beneficial vascular effects of antidepressants, irrespective of the underlying course of depression.

## 1. Introduction

Depression and cardiovascular disease (CVD) are currently the most common causes of disability in high-income countries [1]. Accumulating evidence indicates that there is a link between the two diseases. Patients with CVD have a higher depression prevalence in comparison with the general population [2]. Inversely, depression increases the risk of new CVD and is associated with worse cardiovascular outcomes [3]. Indeed, there is an association between depression and CVD risk factors such as hypertension, diabetes mellitus, smoking, and hypercholesterolemia [4,5] as well as with endothelial dysfunction and arterial stiffness, which predispose a person to increased CVD and mortality [6,7]. On the other hand, there is conflicting evidence on the effect of antidepressant treatment in reducing CVD risk. In patients with acute coronary syndrome and depressive symptoms, antidepressant treatment may reduce CVD risk [8], but evidence is incongruent [9]. Furthermore, in patients suffering from depression with or without stable CVD, evidence is either lacking or suggests no beneficial effects [10]. Due to these discrepancies, international guidelines for CVD prevention recommend depression as a risk enhancer of CVD but do not provide a definitive recommendation on the treatment of depression to reduce cardiovascular events [11]. Moreover, there are no specific recommendations on which class of antidepressants should be prescribed for a reduction in CVD risk due to a lack of convincing evidence [8,9]. Understanding the effect and mode of action of antidepressant classes on cardiovascular function will shed more light on the underlying mechanisms associating antidepressant treatment with CVD. Indeed, accumulating evidence indicates that different classes of antidepressants have off-target effects in the early stages of vascular dysfunction and atherosclerosis, which forms the basis for CVD development. In this review, the currently available data on these off-target arterial effects of antidepressants are summarized and critically discussed, aiming to discriminate between antidepressants with definite beneficial and detrimental actions on markers of early arterial disease. Furthermore, we conclude that the evidence supporting the hypothesis that antidepressants exert beneficial effects on the cardiovascular system may be attributed not only to the antidepressant class but also to the pattern of response of depressive symptoms to treatment.

## 2. Markers of Subclinical Arterial Disease and Their Clinical Relevance in Depression

### 2.1. Search Strategy

**Figure 1:**
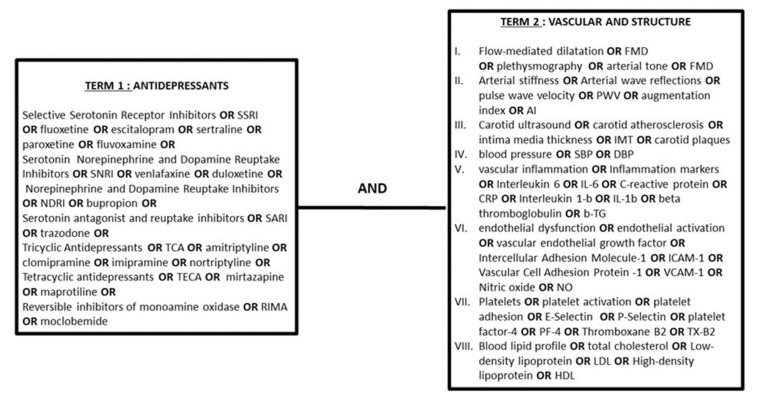
Search strategy depicting the terms and their combinations used in PubMed.

We, hereby, present our search strategy for all databases (Figure 1) as well as the se-lection criteria set for data reporting in compliance with the PRISMA guidelines [12]. This review incorporated the results of searches until the 30th of March 2021 conducted on the electronic bibliographic databases PubMed and Google Scholar.

### 2.2. Selection Criteria for Studies in This Review

Studies included in this review were required to describe the effect of at least one class of antidepressants on clinical and molecular markers of subclinical atherosclerosis and endothelial function as well as on the progression of atherosclerosis in experimental models and human studies. In vivo, ex vivo, and in vitro studies were included. Each study was evaluated by four reviewers who worked independently. Studies that did not meet these criteria were excluded from this review. In total, 30 preclinical (presented in Table 1) and 39 clinical studies (presented in Table 2, Table 3 and Table 4) were included in this review. The total number of participants in clinical studies amounted to 34,773. The studies’ characteristics, number of participants, and findings are presented in separate tables (Table 1, Table 2, Table 3 and Table 4).

Contemporary diagnostic modalities allow us to accurately and non-invasively assess human vascular disease at its early subclinical stages using well-validated imaging and hemodynamic markers. These surrogate markers of CVD risk are applied in research and clinical practice in primary and secondary prevention. A meta-analysis confirmed that depressive symptoms are negatively associated with subclinical arterial disease [13]. Specifically, the following vascular and hemodynamic markers are commonly used as outcome measures in studies exploring the effect of antidepressants on arterial disease. 

### 2.3. Markers of Arterial Wave Reflections and Central Hemodynamics

Increased brachial systolic and diastolic blood pressure (BP) are well-established biomarkers of increased CVD risk [14]. Recent evidence indicates that central (aortic) BP may be a better predictor of future cardiovascular events than peripheral BP [15] because they also reflect the effect of arterial wave reflections from the periphery to the aorta [16]. Augmentation of systolic aortic pressure by the early return of wave reflections due to aortic stiffening and increased peripheral resistance leads to the development of hypertension [17], increased cardiac afterload, and lower diastolic coronary flow [18]. Aortic blood pressure, arterial wave reflections, and aortic stiffness can be readily calculated non-invasively by applanation tonometry using pressure waveforms recorded from superficial sites distal to the aorta and calibrated to the peripheral BP [15]. Pulse Wave Velocity (PWV), as a marker of aortic stiffness, and augmentation index (AIx), as a marker of aortic wave reflections, increase with age, and both constitute risk factors for CVD [19,20] and are associated with increased CVD risk. Both PWV and AIx have been found to increase in patients with depression [21,22], but whether they are associated with increased incidence of hypertension and CVD in this population is not known [23].

### 2.4. Markers of Macrovascular and Microvascular Endothelial Function

Flow-mediated dilation (FMD) is an ultrasound-based non-invasive technique, which is considered the gold-standard method to non-invasively assess conduit artery endothelial function. In brief, this technique uses a sphygmomanometer at the level of the antebrachium and post-occlusion reactive hyperemia is induced at the level of the brachial artery. Subsequently, shear stress increases, inducing FMD. In endothelial dysfunction, FMD is decreased. Brachial FMD is an independent predictor of cardiovascular events and all-cause mortality [24]. Strain gauge plethysmography and Laser Doppler Velocimetry (LDV) using direct current iontophoresis are methods used to assess microvascular endothelial function. Depression is associated with impaired endothelial function and worse FMD [25].

### 2.5. Markers of Carotid Subclinical Atherosclerosis

Intima media thickness (IMT) is the thickness of the intimal and medial layers of the artery wall. IMT is measured non-invasively by ultrasound and is considered an established marker of early atherosclerosis [26]. IMT is closely associated with traditional CVD risk factors and with future cardiovascular events [27,28]. The presence of carotid atherosclerotic plaques represent more advanced stages of atherosclerosis than IMT [29] and is also associated with increased mortality and CVD events [30]. Patients with depression present a greater increase in IMT, independently of classic CVD risk factors [31].

## 3. The Effect of Antidepressant Treatment on Arteriosclerotic Processes: Experimental Data

Among experimental studies examining the effect of antidepressants on vascular function, there is wide variability in the design, the drug dosage, and the targets measured. This variability may partly explain some conflicting results in the literature. Overall, as shown in Table 1, there is consistent evidence demonstrating that serotonin reuptake inhibitors (SSRIs) may act beneficially on endothelial function, possibly delaying the atherosclerotic process.

**Table 1:** Experimental studies on antidepressants and vascular function.

Study	Drug/Dose	Treatment Duration	Study Model	Outcome
Matchkov et al., 2015 [32]	Escitalopram 5 mg/kg/d	3 weeks	Male Wistar rats exposed to CMS	↑ Endothelial function in small arteries via ↓ COX-2 dependent relaxation and ↑ endothelium-dependent hyperpolarization-like pathways
Unis et al., 2014 [33]	Escitalopram Dose NA	6 weeks	Male albino Wistar rats on HFD	↓ Atherosclerotic changes, together with a significant ↓ in VCAM-1 expression in abdominal aortic endothelium and ↓ TG, TC, and LDL
Lopez-Vilchez et al., 2016 [34]	Escitalopram 28 mg/d	24 weeks	HUVEC exposed to MD patients’ whole blood serum	↓ ICAM-1 and oxidative stress (with ↑ presence of eNOS and ↓ ROS production)
Bruno V D Marques et al., 2017 [35]	Fluoxetine 5 mg/kg/d	28 h	Wistar rats	↓ Aortic relaxation to a single restraint test in rat offspring
Janaina A Simplicio et al., 2015 [36]	Fluoxetine 10 mg/kg/d	21 days	Male Wistar rats	↑ Thoracic aorta vasoconstriction by phenylephrine, ↑ BP and PGF2a, and ↓ nNOS
Camila A Pereira et al., 2015 [37]	Fluoxetine 10 mg/kg/d	21 days	Wistar rats	↑ Endothelium-dependent and -independent relaxation of mesenteric resistance arteries via ↑ eNOS activity, NO generation, and KCa channel activation
Dan Dan-Han et al., 2012 [38]	Fluoxetine 10 mg/d	21 days	Adult male Sprague Dawley rats	↓ ROS generation, pulmonary artery pressure, and HIF-1 and VEGF production
Mohamed Habib et al., 2015 [39]	Fluoxetine 10 mg/kg/d	21 days	Male Wistar rats diabetes induced, under CMS	↓ Aortic expression of IL-1β and TNF-a; ↓ BP; ↓TG, TC, and LDL
Isingrini et al., 2011 [40]	Fluoxetine 10 mg/kg/d	5 weeks	Male DBA/2 J mice subjected to CMS	↔ MMP-9, PAI-1, VCAM-1, and ICAM-1 expression
M. Rami et al., 2018 [41]	Fluoxetine 18 mg/kg/d	16 weeks	Apo-E-deficient mice	↑ Atherosclerotic lesions of carotid arteries, ↔ TC
Isingrini et al., 2012 [42]	Fluoxetine 10 mg/kg/d	20 weeks	BALB/c mice subjected to CMS	↓ CMS detrimental effect on NO-related endothelial-dependent relaxation of aortic rings
Tsai et al., 2014 [43]	Fluoxetine/Bupropion/Imipramine/Moclobemide/Venlafaxine/Mirtazapine 10^−8^–10^−5^ M	-	LPS-activated THP-1 human monocytes	Fluoxetine and bupropion ↓ LPS-induced IP-10 expression
Domokos Gero et al., 2013 [44]	Paroxetine 10 mg/kg/d	28 days	BEnd.3 murine cells, EA hy926 human endothelial cells, and Male Sprague Dawley rats induced with diabetes	↑ Acetylcholine-induced rat aortic relaxation, ↓ mitochondrial ROS production in endothelial cells
Laleh Rafiee et al., 2016 [45]	Fluvoxamine 10^−8^ M–10^−6^ M	-	LPS-stimulated human endothelial cells	↓ ICAM-1, VCAM, COX2, and iNOS expression
Lekakis et al., 2010 [46]	Fluvoxamine/Sertraline 10^−7^ M–10^−4^ M	3 months	HAEC	↓ U937 cell adhesion to TNFa-stimulated HAECs, ↓ VCAM-1 and ICAM-1 expression
Silverstein Metzler et al., 2017 [47]	Sertraline 20 mg/kg	18 months	Female cynomolgus monkeys	↑ CAA measured via histomorphometry
Shively et al., 2015 [48]	Sertraline 20 mg/kg	18 months	Female cynomolgus monkeys	↑ CAA measured via histomorphometry, ↔ plasma lipids
Maes et al., 1999 [49]	Sertraline 10^−6^, 10^−8^/Clomipramine 10^−6^, 10^−9^/ Trazodone 10^−6^, 10^−8^	-	Whole blood of healthy human subjects (9)	All ↓ IFNγ, clomipramine, and sertraline; ↑ IL-10
J M Vila et al., 1999 [50]	Sertraline/Nortriptyline/Amitriptyline 3 × 10^−7^–10^−4^ m	-	Human mesenteric arteries	Sertraline, amitriptyline, and nortriptyline ↓ human artery contraction
Joost P van Melle et al., 2004 [51]	Sertraline 0.1–300 μmol/L	-	Pre-contracted rat aortae, HIMA	↑ Endothelial-independent vascular dilation in pre-contracted vessels
Prabhat Singh et al., 2016 [52]	Venlafaxine dose NA	-	Adult male Wistar rats	↑ Endothelial function, assessed by means of a BIOPAC system
S Ribback et al., 2012 [53]	Venlafaxine/Fluoxetine/Tranylcypromine/Amitriptyline (0.05–500 μM)	-	Rat aortic ring dilation after precontraction with phenylephrine	↑ Aortic relaxation in all except for venlafaxine, which promoted contraction
Qinghua LV et al., 2014 [54]	Venlafaxine 10^−8^, 10^−5^ M	20 min	HBMEC	Protection against MGO (methylglyoxal)-mediated endothelial cell injury
Hoda I Bahr et al., 2019 [55]	Duloxetine 15–30 mg/kg	13 weeks	Male Swiss albino mice induced with diabetes	↓ VEGF, ↓ iNOS expression
Brustolim et al., 2006 [56]	Buproprion 100 mg/kg	90 min	LPS-induced male 6-week-old BALB/c mice	↓ Serum TNF-α, IL1-β, IFN-γ, and NO; ↑ in IL-10
Mai Ahmed et al., 2014 [57]	Buproprion 50 kg	4 weeks	Wistar male rats on HFD	↓ Serum TNFa, with no effect seen on aortic IMT or aortic response to acetylcholine, ↔ TG
Labib et al., 2019 [58]	Imipramine 20 mg/kg/day	2 weeks	Male Wistar rats exposed to CMS and HFD	↔ Imipramine on aortic histological abnormalities, and level of CEPCs and VEGFR-2
Ismail et al., 2014 [59]	Imipramine 20 mg	3 weeks	Male Wistar rats	↓ Endothelium-dependent relaxation of the thoracic aorta, ↓ TNF-a expression with imipramine
Rodica Lighezan et al., 2016 [60]	Moclobemide/Clorgyline/Selegiline 10 μmol/L	30 min	HIMA (human internal mammary arteries)	↑ Endothelium-dependent relaxation
Laleh Rafiee et al., 2016 [61]	Maprotiline 10^8^, 10^−6^	-	LPS-stimulated HUVEC	↓ VCAM-1 and ICAM-1 expression

BP: Blood Pressure, CAA: Carotid Artery Atherosclerosis, CMS: Chronic Mild Stress, CEPC: Circulating Endothelial Progenitor Cells, cGMP: Cyclic Guanosine Monophosphate, COX-2: Cyclo-oxygenase-2, HFD: High Fat Diet, HAEC: Human Aortic Endothelial Cells, HBMEC: Human Brain Microvascular Endothelial Cells, HIMA: Human Internal Mammary Arteries, HUVEC: Human Umbilical Vein Endothelial Cells, HIF-1: Hypoxia-Inducible Factor, ICAM: Intercellular Adhesion Molecule, IFN: Interferon, IP-10: Interferon Gamma Inducible Protein, IMT: Intima-Media Thickness, IL: Isoleucine, LPS: Lipopolysaccharide, LDL: Low-Density Lipoprotein, MD: Major Depression, MMP: Matrix Metalloproteinase, NO: Nitrous Oxide, e/i/nNOS: Endothelial/Inducible/Neuronal Nitric Oxide Synthase, PAI: Plasminogen Activator Inhibitor, PGF2a: Prostaglandin F2a, ROS: Reactive Oxygen Species TNF: Tumor Necrosis Factor, TC: Total Cholesterol, TG: Triglycerides, VCAM: Vascular Cell Adhesion Molecule, VEGF: Vascular Endothelial Growth Factor, VEGFR: Vascular Endothelial Growth Factor Receptor. ↑: Increase, ↓: Decrease, ↔: No Effect, NA: Not Available.

### 3.1. Serotonin Reuptake Inhibitors (SSRIs)

SSRIs inhibit the reuptake of serotonin (5-hydroxytryptamine (5-HT)) by blocking the serotonin transporter and thus enhancing serotoninergic neurotransmission [62].

#### 3.1.1. Fluoxetine

Most evidence converges towards a beneficial effect of fluoxetine on endothelial function. In a recent study [39], male Wistar rats were induced with diabetes and subjected to chronic stress. Fluoxetine treatment decreased the expression of the pro-inflammatory and pre-atherosclerotic cytokines tumor necrosis factor-α (TNF-α), interleukine-1b (IL-1b), and Toll-like receptor 4 (TLR-4). TNF-α is a cytokine that is derived from endothelial and smooth muscle cells and macrophages associated with coronary atheroma and is often elevated in the presence of depression [63]. Furthermore, fluoxetine reversed chronic stress and diabetes-induced endothelial impairment, a conclusion drawn from ex vivo thoracic aorta relaxation measurements. In another study in male Wistar rats [37], chronic fluoxetine treatment for 21 days increased the vascular relaxation of mesenteric arteries through both an endothelium-dependent and an endothelium-independent mechanism. Specifically, fluoxetine augmented the phosphorylation of endothelial nitric oxide synthase (eNOS), the endothelium-derived enzyme mediating the production of nitric oxide (NO), NO production, and subsequent vascular relaxation. NO is a molecule that is synthesized by a family of enzymes that are known as NO synthases (NOS) from the amino-acid L-arginine [64]. It induces vasorelaxation and inhibits proatherogenic conditions such as the oxidation of low- density lipoproteins, smooth muscle proliferation and migration, and platelet proliferation and adhesion [65]. The increased activity of potassium channels induced by fluoxetine was also found to be an endothelium-independent mechanism of vascular relaxation. In contrast, fluoxetine for 21 days induced endothelial dysfunction in the rat aortas of male Wistar rats [36] via vasoconstriction, increased BP, enhanced the expression of Prostaglandin F2a (PGF2a), and reduced neuronal nitric oxide synthase (nNOS) production. These results suggest that, although fluoxetine may improve endothelial function, other endothelial-independent mechanisms such as nNOS or vasoconstrictor agents may oppose these beneficial effects. To that end, intrauterine exposure of Wistar rats to fluoxetine suppressed the ex vivo aortic hypo-contraction response to stress in offspring [35]. However, as the authors acknowledged, it is unlikely that the observed reduction in vascular relaxation by fluoxetine was due to endothelial dysfunction because the aortic responses to acetylcholine and phenylephrine were preserved when compared with that in the controls. In support of the beneficial rather than detrimental effects of fluoxetine on endothelial function, fluoxetine improved endothelium-dependent relaxation of the thoracic aorta through increased NO production in a study of BALB/c mice subjected to chronic stress for 7 weeks [42]. Similarly, in a study performed on aortic rings from Lewis A1 rats exposed to various antidepressants [53], fluoxetine promoted vascular relaxation through the endothelial-dependent NO-cyclic guanosine monophosphate (cGMP) pathway mechanism. Finally, in a study where lipopolysaccharide (LPS)-induced THP-1 human monocytes were exposed to high doses of fluoxetine, the expression of the interferon γ-inducible protein 10 chemokine (IP-10), a potentially pre-atherosclerotic chemokine, was suppressed [43]. On the other hand, in a study including apolipoprotein E-deficient mice [41], fluoxetine promoted the formation of atherosclerotic lesions. However, in vitro exposure of murine blood to fluoxetine enhanced leukocyte adhesion only under the presence of inducing chemokine (C-C motif) ligand 5 (CCL5), a potent pro-atherosclerotic chemokine promoting vascular inflammation [66]. These findings suggest that, although fluoxetine may directly act beneficially on endothelial function, its effects may be negated or reversed under conditions of vascular inflammation, a pivotal mechanism of atherogenesis. Along these lines, in a study involving non-atherogenic DBA/2 J mice subjected to chronic stress [40], fluoxetine did not alter the expression of adhesion molecules intercellular adhesion molecule 1 (ICAM-1) and vascular cell adhesion protein 1 (V-CAM-1), coagulation factor plasminogen activator inhibitor-1 (PAI), and metallopeptidase 9 (MMP-9).

#### 3.1.2. Escitalopram

Experimental data investigating escitalopram consistently indicate a beneficial effect on endothelial function. In a study involving male Wistar rats exposed to chronic stress for 8 weeks, escitalopram reduced oxidative stress and increased vascular relaxation in small mesenteric arteries via an NO-independent and endothelium-dependent hyperpolarization (EDH) mechanism and via a cyclooxygenase (COX-2)-related pathway [32]. The lack of an NO-mediated action by escitalopram may be attributed to the less potent impact of NO-mediated relaxation in smaller vessels such as mesenteric arteries compared with the aorta [32]. The antioxidant effect of escitalopram was mediated by the activation of antioxidant enzymes, such as superoxide dismutase-1, catalase, and glutathione peroxidase, in male Wistar rats on a high fat diet treated with escitalopram [33]. Escitalopram also reduced the expression of vascular cell adhesion protein-1 (V-CAM-1) by mitigating the production of transcription factors nuclear factor kappa-light-chain-enhancer of activated B cells (NF-κB) and activator protein-1, leading to less severe aortic atherosclerosis lesions. In support of these observations, exposure of human umbilical vein endothelial cells (HUVECs) to serum from patients with depression treated for 24 weeks with escitalopram resulted in the reduced expression of I-CAM compared with those at baseline [34].

#### 3.1.3. Sertraline

Current evidence suggests a mitigating effect of sertraline on atherosclerosis. Sertraline reduced the expression of the pro-inflammatory cytokine interferon-γ (IFN-γ) and increased the expression of the anti-inflammatory and anti-atherogenic cytokine interleukin-10 (IL-10) in stimulated human whole blood with LPS and phytohemagglutinin [49,67]. Similarly, in human aortic endothelial cells (HAEC) stimulated with TNF-α, sertraline reduced the expressions of VCAM-1 and ICAM-1 and inhibited the adhesion of HAECs to U937 monocytes. It is suggested that sertraline binds to cell surface receptor molecules coupled to intracellular calcium transients, thereby inducing the expression of constitutive nitric oxide synthase (cNOS) and NO production, which then stabilizes NF-κB [46]. Sertraline’s vascular effects may also be mediated by endothelium-independent mechanisms through the inhibition of calcium entry in smooth muscle cells [50,51]. Surprisingly, in two experimental models with female cynomolgus monkeys [47,48], sertraline treatment for 18 months promoted carotid artery atherosclerosis only in depressed animals when compared with non-depressed animals. These findings suggest that depression is a modulating factor for sertraline’s action on atherosclerosis, which merit further investigation in conjunction with the evidence presented above that demonstrate the detrimental effects of sertraline under pro-inflammatory conditions.

#### 3.1.4. Paroxetine

One study demonstrated paroxetine’s ability to reduce mitochondrial and cytosolic reactive oxygen species (ROS) production and to protect against mitochondrial and nuclear DNA damage in hyperglycemia-induced Bend3 murine and hy926 human endothelial cells [44]. Although the mechanism was unclear, the antioxidant effects of paroxetine appear to require the sesamol moiety of paroxetine. Importantly, paroxetine also salvaged the endothelial-dependent aortic response to acetylcholine in Sprague Dawley diabetic rats [44].

#### 3.1.5. Fluvoxamine

Fluvoxamine reduced the expression of adhesion molecules I-CAM-1 and V-CAM-1 in human aortic endothelial cells (HUACs) stimulated by TNF-α, with a mechanism similar to that of sertraline’s [46]. Similarly, fluvoxamine reduced the expression of the adhesion molecules V-CAM-1, I-CAM-1, COX-2, and inducible nitric oxide synthase (iNOS) in HUACs induced with LPS and suppressed their local expression after carrageenan injection in male Wistar rats through an NF-KB-dependent mechanism [45].

#### 3.1.6. The Effect of SSRIs on Lipid Metabolism

The scarce experimental data from preclinical models suggest that SSRIs have either a lowering or a neutral effect on cholesterol and lipoproteins. Specifically, in a model including male Wistar rats on a high fat diet, the use of escitalopram reduced the levels of triglycerides (TG), total cholesterol (TC), and low density lipoprotein (LDL) [33] and fluoxetine also reduced the levels of TG, TC, and LDL in male Wistar rats exposed to chronic restraint stress [39]. Moreover, in female adult cynomolgus macaques, sertraline had no effect on plasma lipids in an 18-month period [48], while fluoxetine did not alter TC in a model including apolipoprotein E-deficient mice on a high fat diet [41]. Finally, in male Wistar rats on a high fat diet, buproprion reduced the G levels [57].

### 3.2. Serotonin and Norepinephrine Reuptake Inhibitor (SNRIs)

SNRIs act by blocking the reuptake of both neurotransmitters serotonin and norepinephrine (NE), resulting in increased neurotransmission and sympathetic activity [68].

#### 3.2.1. Venlafaxine

Experimental data on venlafaxine’s effects on vascular integrity are conflicting. In albino Wistar rats with provoked renovascular hypertension [52], venlafaxine improved endothelial function, increased NO production, and reduced oxidative stress. In another study, exposure of human brain microvascular endothelial cells (HBMEC) to venlafaxine [54] attenuated MGO (methyglyoxal)-mediated endothelial injury. In contrast, a high of dose venlafaxine enhanced endothelium-intact aortic ring contraction in Lewis 1A rats through the induction of endothelium-derived vasoconstrictive agents, including endothelin-1, angiotensin II, and prostaglandins [53].

#### 3.2.2. Duloxetine

Limited data suggest an anti-inflammatory effect of duloxetine in diabetic retinopathy. Duloxetine downregulated the expression of iNOS, vascular endothelial growth factor (VEGF), and transforming growth factor beta (TGF-β) in the retina of diabetic male Swiss rats [55].

### 3.3. Norepinephrine–Dopamine Reuptake Inhibitors (NDRIs)

NDRIs act by blocking the reuptake of both neurotransmitters norepinephrine and dopamine, resulting in increased noradrenergic and dopaminergic neurotransmission.

#### Bupropion

Current evidence suggests that bupropion exerts anti-inflammatory vascular effects, but a corresponding beneficial effect on vascular function and structure has not been demonstrated. Specifically, bupropion reduced the expression of hepatic TNF-α in male Wistar rats on a high fat diet [57] and inflammatory chemokine interferon-γ inducible protein (IP-10) [43] on human THP-1 monocytes induced by LPS in a mitogen activated protein kinase (MAPK)- and NF-κB-independent manner. Similarly, in male 6-week-old BALB/c mice subjected to LPS shock, bupropion decreased the serum levels of TNF-α, IL-1β, and IFN-γ while it increased the expression of the protective IL-10 cytokine [56]. This immunomodulatory behavior of bupropion might be mediated by agonist effects at D1 dopaminergic or beta-adrenoceptors on macrophages and/or lymphocytes, resulting in increased intracellular cAMP and decreased TNF-α synthesis [56]. On the other hand, the expected bupropion-mediated increase in bioavailability of norepinephrine and dopamine may offset its anti-inflammatory properties and explain the lack of beneficial action on thoracic aorta function and structure in male Wistar rats on a high fat diet [57].

### 3.4. Serotonin Antagonist and Reuptake Inhibitors (SARI)

Limited evidence consisting of one study [49] indicates that trazodone reduces the levels of IFN-γ in human whole blood of healthy subjects stimulated with LPS.

### 3.5. Tricyclic Antidepressants (TCAs)

#### 3.5.1. Amitriptyline

Limited evidence indicates that amitriptyline exerts vasorelaxant properties. In aortas from Lewis 1A rats, amitriptyline promoted both endothelium-dependent vasodilation, via increased NO production, and endothelium-independent vasodilation, via I1 adrenoreceptor antagonistic properties [53]. Similarly, in human mesenteric arteries, amitriptyline promoted vasodilation by enhancing α-adrenoreceptor blockade and by inhibiting calcium entry into smooth muscle cells through voltage-dependent calcium channels [50].

#### 3.5.2. Clomipramine

One study reported that clomipramine mitigated the expression of IFN-γ and upregulated IL-10 in LPS-stimulated whole human blood [49]. Clomipramine’s serotonergic activity, i.e., depletion of intracellular 5-HT stores, increased extracellular 5-HT, and 5-HT2A/2C receptor blockade, may account for these effects [49].

#### 3.5.3. Imipramine

Experimental data mainly points to a neutral or even a detrimental effect of imipramine on endothelial function. In Wistar male rats exposed to chronic mild stress (CMS) or a high fat diet, imipramine mitigated the expression of TNF-α in the thoracic aorta but did not delay atherogenesis [58]. In another study, imipramine did not reduce the expression of the chemokine IP-10 and of three mitogen-activated protein kinase (MAPK) pathways, in Th1 LPS-stimulated human monocytes [43]. Importantly, imipramine impaired acetylcholine-mediated aortic relaxation despite decreasing TNF-α mRNA levels in the aorta of male Wistar rats on a high fat diet [59].

#### 3.5.4. Nortriptyline

In human mesenteric arteries, nortriptyline induced direct smooth muscle relaxation via the inhibition of calcium entry and indirect vascular relaxation via the blockade of α-adrenoreceptors and reversal of endothelin-1 activity [50].

### 3.6. Tetracyclic Antidepressants (TECAs)

#### 3.6.1. Mirtazapine and Maprotiline

In LPS-stimulated human monocytes (THP-1 cells), no correlation was found between mirtazapine treatment and the expression of IP-10 chemokine [43]. The treatment of LPS-stimulated HUVECs with maprotiline downregulated the expression of ICAM-1 and VCAM-1 through an NF-κB-dependent mechanism [45]. Maprotiline is also thought to prevent the phosphorylation of p38 MAPK in LPS-stimulated cells [69].

#### 3.6.2. Reversible Inhibitors of Monoamine Oxidase A (RIMAs)

In human internal mammary arteries [60], moclobemide improved endothelium-dependent relaxation, probably via the overproduction of cGMP, normally limited in the presence of monoamine oxidase (MAO), subsequently leading to increased NO availability [70].

## 4. The Effect of Antidepressant Treatment on Arteriosclerotic Processes: Human Molecular Data

It has been proposed that multiple mechanisms of vascular injury and inflammation are induced in patients who suffer from depression. These encompass but are not limited to increased vascular sympathetic tone and vascular resistance due to increased norepinephrine levels, platelet activation, and increased inflammatory burden [71]. There is a wide discrepancy in the literature regarding the effect of antidepressants on these pro-atherosclerotic mechanisms. Table 2 depicts the results from prospective clinical studies examining the effect of antidepressants on circulating pro-atherosclerotic markers and hemodynamic markers of sympathetic activity.

**Table 2:** The effect of antidepressants on human molecular markers.

Type of Dysfunction	Study	RX (mg)	Duration	Patient Population	Design	Marker
Autonomous Nervous System	Shores et al., 2000 [72]	Sertraline 50 mg/placebo	2 days	12 healthy controls	OPC	↓ NE
	Barton et al., 2007 [73]	SSRI	12 weeks	39 MDD-76 healthy controls	OPC	↓ SNS activity
Inflammation	Eller et al., 2008 [74]	Escitalopram 10–20 mg/day	12 weeks	100 MDD	RCT	↔ sIL-2R, IL-8, TNF-α
	Blumenthal, 2012 [75]	Sertraline 50–200 mg/d	16 weeks	101 elevated depressive symptoms and ACS	RCT	↔ PF4, CRP, βTG, IL-6
	Pizzi et al., 2009 [76]	Sertraline 70 ± 39 mg and placebo	20 weeks	95 CHD and depression (47 sertraline and 48 placebo)	RCT	↓ CRP, IL-6
Endothelial injury/dysfunction	Nathalie Lara, 2003 [77]	Paroxetine 10 mg/d and 20 mg/d	9 weeks	18 healthy controls	OPC	↑ NO
	M Deuschle, 2015 [78]	Venlafaxine (46 mg)/mirtazapine (215 mg)	4 weeks	86 MDD	RCT	↔ VEGF
	Wendy Chrapko, 2006 [79]	Paroxetine 10 mg/d–20 mg/d	9 weeks	12 MDD and 12 healthy controls	PC	↑ NO plasma levels, ↔ eNOS activitiy
	Dawood et al., 2016 [80]	SSRI	95 days	33 with MDD	OPC	↔ ICAM-1, VCAM-1, P-selectin, NE
	Lopez-Vilchez et al., 2016 [34]	Escitaloprame, average 28 mg/day	24 weeks	12 MDD and 12 healthy controls	OPC	↓ CECs, VWF, VCAM-1; ↑ EPCs
	Lekakis et al., 2010 [46]	Sertraline 50 mg/placebo	3 months	25 MDD-CHF	RCT	↓ VCAM-1, ICAM-1
Platelet activation	N Hergovich, 2000 [81]	Paroxetine 20 mg/d	14 days	16 healthy controls	RCT	↓ intraplatelet serotonin levels, platelet plug formation and responsiveness to thrombin receptor activating peptide
	Hantsoo et al., 2014 [82]	SSRI (scitalopram 20 mg/sertraline 20 mg/fluoxetine 50 mg)	4 weeks	28 MDD/PMDD/PPD	OPC	↔ platelet aggregation, ↓ platelet NO
	Serebruany et al., 2005 [83]	Sertraline	16 weeks	55 MDD	RCT	↓ PF4, PECAM-1, β-TG, P-selectin, TxB2, E-selectin, 6-keto-PGF1a; ↑ VCAM-1
	Serebruany et al., 2003 [84]	Sertraline	24 weeks	64 ACS	RCT	↓ Platelet factor 4, βTG, PECAM-1, P-selectin, TxB2, 6-keto-PGF1a, VCAM-1, and E-selectin
Blood Lipid Profile	Paslakis et al., 2011 [85]	Paroxetine	4 weeks	35 MDD and 35 healthy controls	RCT	↓ Lpa
	Perry et al., 1990 [86]	Trazodone	6 weeks	36 MDD	RCT	↓ TC
	Hummel et al., 2011 [87]	Venlafaxine, mirtazapine	4 weeks	65 MDD and 31 healthy controls	RCT	↓ LDL/HDL ratio in responders

ACS: Acute Coronary Syndrome, βTG: Betathromboglobulin, CRP: C Reactive Protein, CECs: Circulating Endothelial Cells, CHF: Congestive Heart Failure, CHD: Coronary Heart Disease, eNOS: Endothelial Nitric Oxide Synthase, EPCs: Endothelial Progenitor Cells, eVWF: Endothelial Von Willebrand Factor, HDL: High-Density Lipoprotein, ICAM-1: Intercellular Adhesion Molecule 1, IL-6: Interleukin-6, IL-8: Interleukin-8, Lpa: Lipoprotein a, LDL: Low-Density Lipoprotein, MDD: Major Depressive Disorder, NE: Norepinephrine, NO: Nitric Oxide, OPC: Observational Prospective Cohort, PECAM-1: Platelet Endothelial Cell Adhesion Molecule-1, PF4: Platelet Factor 4, PPD: Postpartum Depression, PMDD: Premenstrual Dysphoric Disorder, PC: Prospective Cohort, RCT: Randomized Controlled Trial, sIL-2R: Soluble Interleukin-2 Receptor, SNS: Sympathetic Nervous System, TC: Total Cholesterol, TxB2: Thromboxane B2, TNF-a: Tumor Necrosis Factor Alpha, VCAM-1: Vascular Cell Adhesion Protein, VEGF: Vascular Endothelial Growth Factor. n↑: Increase, ↓: Decrease, ↔: No Effect.

### 4.1. Inflammation Markers

#### 4.1.1. SSRIs

In a retrospective study [88] evaluating the effect of various classes of antidepressants on the expression of inflammatory markers in 2981 depressed and healthy subjects, there was a reduction in serum IL-6 in men suffering from depression treated with SSRIs.

In a randomized controlled trial (RCT) examining 95 coronary heart disease (CHD) patients with depressive symptoms, sertraline treatment for 20 months of reduced serum levels of C-reactive protein (CRP) and IL-6 [76]. In contrast, in another RCT examining 101 patients with Acute Coronary Syndrome (ACS) and depressive symptoms (>7 on the Beck Depression Inventory), sertraline treatment at a dosage of 50–200 mg/day for 16 weeks did not affect the serum levels of platelet factor 4 (PF4), IL-6, beta-thromboglobulin (βTG), and CRP, compared with the placebo [75]. The observed lack of effect in these patients may be attributed to the potent anti-inflammatory effect of statins that are prescribed in all ACS patients after an ischemic event. A meta-analysis, including 22 studies, concluded that SSRIs contribute to a reduction in the expression of inflammatory cytokines IL-1β and possibly IL-6 in patients with depression [89]. It was also noted that the proportion of regulatory T-lymphocytes (Tregs) increased in treated patients. As Tregs suppress innate immunity, this may be a mechanism through which antidepressant treatment reduces IL-1β levels. Conversely, it is possible that reduced levels of IL-1β may permit differentiation of Tregs. Other inflammatory cytokines such as TNF-α were not associated with SSRI treatment despite an improvement in depressive symptomatology.

#### 4.1.2. Non-SSRIs

There is a lack of RCT data studying the role of SNRIs in vascular inflammation in humans. In a retrospective study [88] involving 2981 individuals with or without depression, SNRIs, TCAs, and TECAs were associated with higher CRP and IL-6 serum levels in patients with depression. These classes of antidepressants possess noradrenergic properties compared with the SSRIs’ exclusive serotonergic effects, possibly promoting inflammation and cytokine production as well as increased BP and metabolic activity [90]. Importantly, in a meta-analysis discussed above, comprising 22 studies, mostly case–control studies, venlafaxine and duloxetine were associated with increased serum levels of TNF-α and IL-6, respectively [89]. Nonetheless, it should be noted that non-SSRI medications are mainly indicated in refractory MD, which is associated with higher inflammatory burden. RCTs are needed to elucidate this issue.

### 4.2. Markers of Endothelial Dysfunction

#### 4.2.1. SSRIs

There is consistent evidence that SSRIs improve markers of endothelial function. In a non-interventional case–control study of 12 patients with depression and 12 healthy controls, with measurements at baseline, week 8, and week 24, following 24 weeks of treatment [34], escitalopram was associated with progressively decreased blood levels of VCAM-1 and Von Willebrand factor and increased circulating endothelial progenitor cells, mediators of vascular repair [34]. Moreover, in an interventional study [79] examining 17 patients with depression and 12 healthy controls, paroxetine was associated with increased serum NO levels. Importantly, NO levels returned to baseline levels after the discontinuation of treatment and did not depend on the level of response to treatment. Thus, a direct effect of paroxetine on endothelial function, irrespective of the course of depressive symptoms, is suggested. These findings were confirmed in a study of 18 healthy males, in whom treatment with paroxetine for 8 weeks, was associated with increased serum NO levels and endothelium eNOS activity, which returned to baseline after discontinuation of treatment [77]. Finally, in an RCT of 25 chronic heart failure patients with depression, sertraline administered at 50 mg/d for 3 months reduced the serum levels of ICAM-1 and VCAM-1 compared with the placebo [46]. Overall, human molecular studies point to a beneficial effect of SSRIs on endothelial function.

#### 4.2.2. Non-SSRIs

Data from one RCT including 47 patients with depression who were randomized to receive venlafaxine, mirtazapine, or placebo indicated that serum VEGF levels were not affected in any treatment group. VEGF is a cytokine that has a mitogenic effect on endothelial cells [91] and, thus, improves endothelial function by promoting endothelial cell regeneration and by impeding thickening of the media [78,92].

### 4.3. Platelet Activation

There is convincing and consistent evidence demonstrating that SSRIs attenuate platelet activation and adhesion. In two RCTs involving post ACS patients, sertraline treatment for 16 and 24 weeks reduced the levels of TGβ, E- and P-Selectin, PF-4, and thromboxane B2 serum [83,84]. These molecules are critically involved in initiating platelet activation and adhesion [93,94]. Interestingly, these effects were not masked by potent concomitant anti-platelet medication administered to these patients. SSRIs halt platelet activation by binding to serotonin transporters on platelets, modifying platelet function through pathways distinct from those affected by anti-platelet medication. Accordingly, paroxetine decreased intraplatelet serotonin content, thereby reducing platelet plug formation under shear stress in healthy male individuals in an RCT [81].

### 4.4. Blood Lipid Profile

#### 4.4.1. SSRIs

There is conflicting evidence regarding the effect of SSRIs on total serum cholesterol and individual atheroclerotic lipoprotein levels. Some observational studies denote a detrimental effect of SSRIs on blood lipid profile as evinced by increased levels of LDL, TC, and TG in the blood of patients receiving this treatment [95,96,97,98]. Conversely, in a small RCT comprising 35 patients with depression and 33 healthy individuals, treatment with paroxetine but not amitryptiline for 4 weeks reduced lipoprotein(a) (LPa) levels [85]. Overall, in a systematic review [99], SSRIs had a statistically nonsignificant effect on TG and TC in patients with depression. The discrepancy between the experimental data and some of the clinical evidence may be due to remission of the depressive state, which is correlated with low cholesterol levels in certain clinical studies [100] and merits further elucidation.

#### 4.4.2. Non SSRIs

Limited data exist on the effect of non-SSRIs on blood lipid profile. In a systematic review [99], the use of TCAs and mirtazapine was related to increased LDL and TC, duloxetine and venlafaxine with reduced HDL, and buproprion with decreased LDL. The adverse role of mirtazapine was further highlighted in another interventional study [101] comprising 50 healthy subjects who displayed increments in TG and TC after 4 weeks of treatment although LDL values were not affected. Conversely, in an RCT including 65 patients with depression [87], venlafaxine and mirtazapine improved the LDL/HDL ratio only in responders. Finally, in an RCT [86] including 36 patients with depression, treatment with trazodone resulted in a reduction in TC after six weeks.

Overall, clinical studies, including a limited number of RCTs consistently demonstrated that SSRIs improve markers linked to endothelial dysfunction, vascular inflammation, and platelet function. On the other hand, they probably aggravate the blood lipid profile. Nonetheless, more randomized control studies are required to confirm and clarify the clinical applicability of these findings. Moreover, the dependence of these effects on the response to antidepressant treatment also needs further exploration, as discussed below.

## 5. The Effect of Antidepressant Treatment on Arteriosclerotic Processes: Clinical Evidence

### 5.1. Blood Pressure and Arterial Wave Reflections

#### 5.1.1. SSRIs

There are several randomized controlled and prospective observational trials that have examined the effect of SSRIs on the BP of patients with depression, demonstrating conflicting results [102,103,104] (Table 3). According to two large meta-analyses, SSRIs do not significantly affect BP. Zhong et al. included 23 RCTs with 13,385 participants in total and concluded that, when compared with placebo, SSRIs did not cause any statistically significant changes to systolic BP (SBP) and diastolic BP (DBP). Moreover, there was no significant difference among the five main SSRI drugs [105]. Similarly, another meta-analysis conducted by Thase et al., which studied the cardiovascular safety of escitalopram, showed that it had no clinically meaningful effect on BP [106]. These negative findings may be attributed to a lack of consideration for the post-treatment depression status. To this end, we observed that aortic and peripheral BP, and augmentation index decreased only in patients with depression who received treatment and experienced an improvement in their depressive symptoms. In this observational study, all patients received a combination of citalopram and risperidone. Interestingly, this finding was in contrast with the increases observed in the same hemodynamic parameters as those who neither received treatment nor experienced an improvement in their symptoms over a period of 6 months [102]. Thus, a beneficial effect of SSRIs on BP should be further considered in RCTs comparing patients according to treatment response. Finally, research should also focus on the effect of SSRIs on aortic BP and aortic wave reflections. Aortic hemodynamics provide additional mechanistic and prognostic information over peripheral BP [107] and may respond differently to interventions [108,109].

**Table 3:** The effects of antidepressants on vascular markers: Randomized Controlled Trials (RCTs) and Observational Prospective Cohort Studies (OPCs).

Study	Depression	CVD	N	Age	Women	Rx	Res	Tx (Weeks)	Study	En. F.	A. S.	B. P.
Derby et al., 2007 [110]	No	No	70	19–74	100%	SNRI (Duloxetine)		3	RCT			↑
Diaper et al., 2013 [111]	No	No	54	23 ± 5	46%	SNRI (Venlafaxine)		3	RCT			↑
Martins et al. 2009 [112]	No	No	24	41 ± 7	50%	NDRI (Bupropion)		1	RCT			↑
Thase et al., 2008 [113]	No	No	300	44 ± 13	39%	NDRI (Bupropion)		4	RCT			↔
Dawood et al., 2016 [114]	Yes	No	31	44 ± 2	61%	SSRI (Various)		24	OPC	FMD ↔		
Hantsoo et al., 2014 [82]	Yes	No	27	38 ± 8	100%	SSRI (Various)		4	OPC	FMD ↔		
Kokras et al., 2019 [102]	Yes	No	37	51 ± 13	30%	SSRI (Citalopram)	24	26	OPC	FMD ↑	PWV ↓, AI ↓	↓
Oulis et al., 2010 [115]	Yes	No	40	57 ± 10	100%	SSRI (Various)	12	6	OPC		PWV ↓	
Peixoto et al., 2018 [103]	Yes	No	30	57 ± 6	76%	SSRI (Escitalopram)		8	RCT			↔
Scuteri et al., 2013 [104]	Yes	No	21	77 ± 4	76%	SNRI (Duloxetine)		52	RCT		PWV ↑	↑
Scuteri et al., 2013 [104]	Yes	No	27	77 ± 5	85%	SSRI (Escitalopram)		52	RCT		PWV -	↑
Tudoran et al., 2019 [116]	Yes	No	128	48 ± 6	60%	SSRI (Sertraline)		26	OPC	IMT↓	PWV ↓	
Blumenthal et al., 2012 [75]	Yes	Yes	64	63 ± 11	30%	SSRI (Sertraline)		18	RCT	FMD ↔		
Pizzi et al., 2009 [76]	Yes	Yes	95	57 ± 8	50%	SSRI (Sertraline)		20	RCT	FMD ↑		

AI: Augmentation Index; FMD: Flow-Mediated Dilation; IMT: Intima Media Thickness; NDRI: Norepinephrine and Dopamine Reuptake Inhibitor; OPC: Observational Prospective Cohort Study; PWV: Pulse Wave Velocity; RCT: Randomized Controlled Trial; SSRI: Selective Serotonin Receptor Inhibitors; SNRI: Serotonin, Norepinephrine, and Dopamine Reuptake Inhibitors; Res: Responders; En. F: Endothelial Function; A. S.: Arterial Stiffness; B. P.: Blood Pressure; ↑: Increase; ↓: Decrease; ↔: No Effect.

#### 5.1.2. SNRIs

There is adequate evidence supporting that SNRIs cause a clinically meaningful increase in blood pressure. In a meta-analysis of eight double-blind placebo-controlled clinical trials, Thase et al. showed that treatment with duloxetine at therapeutic doses of 40–120 mg/d led to increases in SBP but not DBP compared with the placebo. This increase, however, was not dose-related and few patients had clinically significant increases [117]. In contrast, venlafaxine presented a dose-dependent augmenting effect on blood pressure [111]. In a recent large meta-analysis, Zhong et al. compared changes in BP when treated with SSRIs versus SNRIs. They concluded that SNRIs lead to a statistically significant increase in BP, compared with SSRIs [105]. The dominant mechanism for this effect may be associated with the SNRI-mediated potentiation of noradrenergic activity [68], possibly explaining discrepancies between venlaflaxine and duloxetine; venlafaxine has a dose-dependent effect on BP due to the fact that it inhibits NE reuptake only at higher doses, while duloxetine prevents the reuptake of both 5-HT and NE at all doses [118].

#### 5.1.3. TCAs

Treatment with TCAs is well known to have significant cardiovascular side effects, with the most common being orthostatic hypotension [119]. This side effect is especially severe in patients with underlying CVD. Interestingly, imipramine caused an orthostatic drop in BP, even at lower than usual therapeutic doses [120]. Orthostatic hypotension caused by TCAs was attributed to their α-adrenergic blocking effects. However, there is some recent evidence linking TCAs to a greater incidence of hypertension [121,122], but clinically evident hypertension caused by TCAs is rare. This phenomenon may be attributed to their effect on vagal tone, as indicated by the reduction in heart rate variability and baroreflex sensitivity [114].

#### 5.1.4. Other Antidepressants

MAOIs are known to cause both orthostatic hypotension and hypertension. They inhibit MAO, causing decreased breakdown of monoamines, resulting in the accumulation of tyramine and increased BP [123]. Orthostatic hypotension is probably due to the gradual displacement of epinephrine from the nerve terminals, which impairs peripheral adrenergic neurotransmission [123]. Bupropion, an NDRI, seems to cause an elevation in BP [112,113]. On the other hand, trazodone, an SARI, is associated with orthostatic hypotension [124]. TECAs, such as mirtazapine, seem to have no effect on BP [125].

### 5.2. Arterial Stiffness

#### 5.2.1. SSRIs

There is only one RCT assessing the effect of SSRIs on arterial stiffness [126]. In this study, escitalopram or duloxetine were randomly assigned to aged patients with depression and PWV was measured before and after treatment. The results were also compared with those in patients without depression who received no antidepressant medication. No effect on PWV was observed after 12 months of escitalopram compared with the non-depressed controls. However, the percentage of treatment response in the escitalopram group and whether there was a difference between responders to escitalopram compared with non-responders were not clarified [104]. To that end, in a non-randomized observational study, we demonstrated an improvement in arterial stiffness in 37 patients with depression [102]. Patients under citalopram at a dose of 20–60 mg and risperidone at a dose of 0.5–1 mg for 6 months improved PWV compared with treatment non-compliant patients who did not complete the protocol. After adjustment for changes in arterial blood pressure and other CVD risk factors known to influence PWV, these differences remained significant. Interestingly PWV was improved only in the group of patients who had a reduced Hamilton Depression Rating Scale (HDRS) score by more than 50%, whereas in those who did not achieve this reduction in HDRS, PWV did not change [102]. Likewise, in a short-term study, we demonstrated an improvement in increased arterial stiffness in 20 women with severe depression after 6 weeks of antidepressant treatment, irrespective of the type of treatment used. Importantly, the magnitude of PWV reduction was directly proportional to clinical improvement [115]. These findings strongly suggest that, in addition to the class of antidepressant drugs, raising the detrimental effect of depression on vascular function may be a pre-requisite to achieving a clinically meaningful benefit for cardiovascular disease. This concept has not been considered in other studies assessing the effect of SSRIs or other antidepressant drug classes on arterial function and may explain some conflicting results [104]. Therefore, future RCTs regarding the effect of SSRIs on arterial stiffness should consider differences between treatment responders and non-responders. Due to the limited data available, it is not possible to conclude whether there are differences between the type of SSRI and drug class, and therefore, further investigation is warranted.

#### 5.2.2. SNRIs

Scuteri et al. found that treatment with duloxetine for 12 months increased PWV by 12% and heart rate by 8.3%. This was independent of blood pressure changes or other traditional cardiovascular factors. Since heart rate may directly affect PWV, this finding may indicate an indirect effect of the drug [104]. SNRIs may also increase arterial stiffness through increased NE availability at the level of arterial wall [127]. Although SNRIs do not appear to increase CVD risk [128], there is a need to further explore the clinical role of the effect of this drug class on markers of arterial stiffness.

#### 5.2.3. Other Antidepressants

No studies have addressed the effect of other anti-depressant drug classes on arterial stiffness.

### 5.3. Endothelial Function

#### 5.3.1. SSRIs

The majority of current evidence indicates a trend for a beneficial effect of SSRIs on endothelial function, but there are also some conflicting results. Pizzi et al. randomized 95 patients with known CVD and depressive symptomatology to sertraline treatment or placebo for 5 months. They found that sertraline treatment improved FMD compared with the placebo [76]. In an observational retrospective study, Sherwood et al. found that patients with CHD and severe depressive symptomatology, predominantly receiving SSRI-based treatment, had better FMD compared with those who received no treatment [71] (Table 4). Similarly, treatment with citalopram for 6 months in patients with depression without CVD improved FMD and PWV only in treatment responders [102]. On the other hand, some studies demonstrated no changes in endothelial function after treatment with SSRIs [75,80,82]. In an observational prospective study, Hantsoo et al. found no improvement in women free of CVD and treated for 4 weeks with paroxetine, fluoxetine, or citalopram. In an RCT in which patients with coronary artery disease (CAD) and depressive symptoms were randomized to receive either sertraline or placebo or exercise, FMD was measured among other markers before and after treatment for 4 months [75]. Although there was no group difference in FMD between combined groups of exercise and sertraline vs. placebo, FMD increased in the sertraline group while it decreased in the placebo group, but a separate statistical analysis for this comparison was not reported. Dawood et al. assessed microvascular endothelial function by strain gauge plethysmography and by acetylcholine iontophoresis (in contrast with FMD, which assesses macrovascular endothelial function) in patients with depression [80]. They found no change in microvascular endothelial function with SSRI treatment. These findings indicate relatively consistent results towards improvement of macrovascular but not microvascular endothelial function in patients diagnosed with major depression. Some discordant findings may be attributed to the fact that the interaction between response to treatment and changes in FMD are not considered in most studies. As described above, paroxetine increased serum NO levels in 17 patients with depression, which returned to baseline after discontinuation independent of response to treatment [79]. However, it seems that, at least in part, SSRIs directly improve endothelial function via off-target effects, irrespective of depression status. Finally, differences in the severity of depression and underlying diseases may also account for this discordance. Therefore, the complex interplay between disease status, antidepressant treatment response, and endothelial function warrants further study, including RCTs and meta analyses.

**Table 4:** The effects of antidepressants on vascular markers: Retrospective Cohort Studies (RCS), Cross-sectional Cohort (CC), and Longitudinal Studies (LS).

Study	Depression	CVD	N	Age	Women	Rx	Res.	Weeks	Study	En. F.	A. S.	B. P.
Sherwood et.al, 2005 [71]	Yes	Yes	143	63 ± 10	31%	Various	-	-	RCS	FMD ↑	-	↑ SBP, DBP (TCAs)
Broadley et al., 2002 [129]	Yes	No	22	18–55	30%	Various	-	-	CC	FMD ↓	-	
Delaney et al., 2010 [122]	Yes	No	622	45–84	41%	Various	-	85	LS		-	
Licht et al., 2009 [121]	Yes	No	20.718	40 ± 12	69%	Various	-	-	RCS		-	
Crookes et al., 2018 [130]	Yes	No	11.183	16–29	47%	Various	-	672	CC		-	↑ SBP, DBP (NS working
Paranthaman et al., 2012 [131]	Yes	No	25	72 ± 5	15%	Various	9	-	RCS	↓ lMT Res > NRes	-	
Camacho et al., 2016 [132]	Yes	No	324	62.1	56%	Various	-	-	RCS	↔ IMT	-	↑ SBP, DBP

CC: Cross-Sectional Cohort, DBP: Diastolic Blood Pressure, FMD: Flow-Mediated Dilation, IMT: Intima Media Thickness, LS: Longitudinal Study, RCS: Retrospective Cohort Study, SBP: Systolic Blood Pressure, Res: Responders, NRes: Non-Responders, En. F: Endothelial Function, A. S.: Arterial Stiffness, B. P.: Blood Pressure, ↑: Increase, ↓: Decrease, ↔: No Effect.

#### 5.3.2. Other Antidepressants

The specific effect of other non-SSRI antidepressant drug classes on endothelial function has not been investigated.

### 5.4. Atherosclerosis of the Carotid Arteries

#### 5.4.1. SSRIs

In an observational prospective study, Tudoran et al. found an improvement in IMT after the administration of sertraline for 6 months to patients with severe or moderate depression and no history of CVD [116]. In a retrospective study, Camacho et al. did not find differences in IMT between patients with a history of previous antidepressant treatment, mostly SSRIs, and those without among a population of 324 patients without CVD [132]. On the other hand, in another retrospective study, Paranthaman et al. found that patients with late-life depression who responded to treatment, mostly SSRIs, had lower IMT than those who did not respond to treatment, that were nevertheless higher than those in the control group without depression [131]. Along these lines and as mentioned above, endothelial function, arterial stiffness, and central and peripheral BP improved only in patients who responded to treatment compared to those with no or minimal change in depression severity following treatment [102]. These findings agree with the hypothesis of an expected higher cardiovascular benefit from antidepressant treatment in patients with depression who respond to treatment and may explain some conflicting results described above, where treatment response was not taken into account. Nevertheless, considering that there are no RCTs examining the effect of SSRIs or other antidepressants on carotid atherosclerosis, no solid conclusions can be deduced from the current observational studies. Thus, current evidence should be built upon by planning new RCT studies comparing the cardiovascular benefit between patients who did and did not respond to antidepressant treatment.

#### 5.4.2. Other Antidepressants

Currently, no studies have been performed examining the effect of non-SSRI antidepressants on carotid atherosclerosis.

## 6. Discussion

Given the close association between depression and depressive symptoms with CVD, it is of great importance to explore available means to reduce CVD risk under these conditions. Thus, understanding the mechanism associated with the effect of antidepressant drugs on the cardiovascular system will provide novel insight into planning new strategies aimed at reducing CV risk in depression. For this purpose, in the current narrative review, we aimed to identify what is currently known regarding off-target effects of antidepressant treatment on vascular function and structure, to highlight gaps in the evidence, and to discuss the future directions pertinent to this field of research.

Based on current evidence, of all antidepressant drug classes, SSRIs possess the most prominent cardiovascular off-target properties, perhaps because they are the most commonly used and studied with recent decades. However, there is considerable conflicting data, disallowing immediate clinical application of these findings. Specifically, SSRIs, in contrast with other antidepressant classes, seem to exert direct protective effects on arterial integrity. Current preclinical experimental evidence indicates that SSRIs reduce vascular inflammation and thrombosis and improve endothelial function, but limited available data do not support a beneficial effect on the development of advanced atherosclerosis. Importantly, results from observational and some RCTs suggest that SSRIs may improve endothelial function and arterial stiffening and may delay the development of carotid atherosclerosis. It should be emphasized that there is accumulating evidence that antidepressant treatment may confer indirect cardiovascular benefits through the improvement of depressive symptoms and the clinical progression from response to remission and finally to recovery from depression. This concept has not been incorporated into RCT designs and may thus mask some beneficial effects of SSRIs on vascular function and structure. Moreover, there is limited data regarding the impact of SSRIs in special populations with depression at high risk for future CVD events, such as patients with diabetes [133], patients with chronic kidney disease [134], and patients with established stroke [135,136] and peripheral artery disease other than CHD and ACS. Despite contemporary risk reduction strategies, CVD risk is still high in these patients [137,138]. For this purpose, it is recognized that there is a need to further reduce this residual risk using novel prevention approaches [139]. Therefore, exploiting off-target beneficial effects of SSRIs, where indicated in this setting, would optimally serve this goal.

Regarding the remaining antidepressant classes, SNRIs seem to act detrimentally on markers of arteriosclerosis due to their noradrenergic properties. Specifically, they increase NE levels, subsequently precipitating cytokine production that leads to an increase in inflammation [68]. Moreover, they are linked to higher BP and increased arterial stiffness. Similarly, TCAs increase vascular inflammation and are also associated with orthostatic hypotension. Overall, experimental and clinical data regarding the non-SSRI antidepressant drug classes are limited and conflicting. Thus, their net effect on vascular health needs to be investigated in experimental models, assessing the development and progression of atherosclerosis, as well as in clinical studies, assessing markers of subclinical atherosclerosis [140].

In conclusion, from a clinical point of view, SSRIs may provide optimal cardiovascular benefit to patients with depression who respond to treatment, i.e., demonstrate symptom reduction. To verify this hypothesis, RCTs are needed to compare changes in markers of vascular function and structure between responders and non-responders to SSRI treatment. Research should also focus on special populations with CVD risk, such as patients with diabetes, patients with chronic kidney disease, and patients with established stroke. Finally, given that specific drug classes such as TCAs and SNRIs deteriorate vascular function and structure, cardiovascular safety of long-term treatment regimens should also be considered a research priority.
